# Eating Compulsivity in Inpatients with Severe Obesity and the General Population: The Italian Version of the Measure of Eating Compulsivity (MEC10-IT)

**DOI:** 10.3390/nu15061378

**Published:** 2023-03-12

**Authors:** Alessandro Alberto Rossi, Giada Pietrabissa, Ashley N. Gearhardt, Alessandro Musetti, Gianluca Castelnuovo, Stefania Mannarini

**Affiliations:** 1 Department of Philosophy, Sociology, Education, and Applied Psychology, Section of Applied Psychology, University of Padova, 35131 Padova, Italy; 2 Interdepartmental Center for Family Research, University of Padova, 35131 Padova, Italy; 3 Clinical Psychology Research Laboratory, Ospedale San Giuseppe, IRCCS Istituto Auxologico Italiano, 28824 Verbania, Italy; 4 Department of Psychology, Catholic University of Milan, 20123 Milan, Italy; 5 Department of Psychology, University of Michigan, Ann Arbor, MI 48109, USA; 6 Department of Humanities, Social Sciences and Cultural Industries, University of Parma, 43121 Parma, Italy

**Keywords:** eating compulsivity, food addiction, binge eating, obesity, eating disorders

## Abstract

This study aimed to validate and investigate the psychometric properties of the Italian version of the Measure of Eating Compulsivity-10 (MEC10-IT) in a sample of inpatients with severe obesity (Study 1), and to test the measurement invariance of the measure across non-clinical and clinical samples (Study 2). In the first study, a confirmatory factorial analysis (CFA) was conducted among 452 patients in order to confirm the factorial structure of the MEC10-IT. In the second study, the psychometric properties of the MEC10-IT were tested on 453 inpatients with severe obesity and a community sample of 311 participants. The CFA confirmed the factorial structure of the MEC10-IT among an Italian sample of adult inpatients with severe obesity (Study 1). The MEC10-IT was also demonstrated to be invariant between the clinical and the community sample and to possess good psychometric properties, as well as excellent screening abilities for classifying individuals with problematic eating behaviors (Study 2). In conclusion, the MEC10-IT could be considered as a valid and reliable tool for the assessment of compulsive eating in both non-clinical and clinical samples and represents a psychometrically sound measure for clinical and research purposes.

## 1. Introduction

Obesity is one of the greatest public health challenges of the 21st century, causing various physical disabilities (e.g., cardiovascular diseases, cancer, diabetes, and musculoskeletal disorders) and psychological problems [1,2]. Worldwide obesity has nearly tripled since 1975 [3], partially due to the increased availability of hyper-palatable and highly processed foods (which are high in fat and sugars with chemical flavors) [4,5,6,7]. Moreover, highly processed foods share the typical characteristics and benchmarks of addiction-target substances—such as tobacco [8].

Indeed, one of the factors explaining the increase in obesity may be food addiction (FA) [9]—a compulsive phenotype that is associated with addiction-like symptoms (e.g., a loss of control, intense craving, and withdrawal) to highly palatable and/or processed food [9,10,11,12,13,14,15,16,17]. Accordingly, converging neurobiological evidence suggests that food overconsumption can trigger the brain reward systems that are implicated in negative reinforcement processes (e.g., reducing negative emotions) similarly to substance-related and addictive disorders (SRAD) [18].

As a result, individuals may become addicted to highly processed foods and thus may be driven to engage in dysfunctional behaviors characterized by an irresistible, compulsive urge to eat highly processed food. More in detail, behavior compulsivity—a persistent drive to enact a behavior (e.g., drug use and food consumption) and the inability to control it—is a central feature of both Substance-Related and Addictive Disorders (SRAD) and behavioral addictions [19,20], as well as of certain eating disorders (ED)—e.g., binge eating disorder (BED) and bulimia nervosa (BN). Indeed, certain food-related psychopathologies, such as FA, BED, and BN, are centrally associated with an irresistible, uncontrollable urge to overeat, despite efforts to control this behavior [21,22]. 

Furthermore, research shows how compulsive (over-) eating behaviors may be dissected into three main facets [20], each of which has analogies with specific aspects of EDs and/or SRADs. The first facet is habitual overeating. Repeated experiences of eating palatable food can establish ordered and structured action sequences so that certain contexts or stimuli that are associated with food easily evocate compulsive overeating. In other words, environmental stimuli associated with food (e.g., the smell of bread, supermarkets, and food commercials) can augment the intense desire for a certain food, as well as “food-seeking” behaviors, regardless of real hunger [23,24,25]. The second facet is related to negative affect. Compulsive eating behaviors often aim to prevent or relieve emotional distress, such as anxiety and sadness, thus triggering a vicious circle between eating and negative affect [26,27,28,29,30]. The third facet is excessive eating despite the awareness that it can lead to several medical conditions that are associated with excessive weight (e.g., cardiovascular disease, type II diabetes, and cancer), as well as psychological issues, psychiatric disorders, and functioning impairment [31,32]. The persistence of compulsive eating behaviors despite their negative consequences is a key point of addictive behaviors with a feeling of a “loss of control”, overeating, and drug taking [33,34,35,36,37,38]. 

Since it was conceptualized as a related, but distinct, construct from that of addiction, much attention has been recently dedicated to defining what constitutes compulsive eating behavior, and how it should be specifically measured and treated [20]. The Brief Measure of Eating Compulsivity (MEC10) is a 10-item instrument that is specifically focused on the evaluation of compulsive eating within the FA framework. Validation of the tool showed excellent test–retest reliability and high internal consistency. Furthermore, scores on the MEC10 were found to be statistically predictive of FA diagnosis, based on SRAD criteria [39].

Since the MEC10 is currently not available for use among the Italian population, the purpose of this two-step study was to examine its psychometric properties. Specifically, a confirmatory factorial analysis (CFA) was employed to investigate the construct validity of the Italian version of the MEC10 (MEC10-IT) in a sample of inpatients with severe obesity (Study 1). Then, the measurement invariance (MI) and other psychometric proprieties of the questionnaire were tested across clinical and non-clinical samples (Study 2). This would allow the scale to be applied to multiple groups of individuals who are (potentially) prone to dysfunctional eating behaviors (such as compulsive eating).

## 2. Study 1: Factorial Structure of the Italian Version of the MEC10

### 2.1. Translation and Cultural Adaptation

According to international guidelines [40,41], the MEC10-IT was first translated from English to Italian by two bilingual translators, independently. A summary version of the tool was then obtained from the independent translations, and back-translated into English by another independent translator whose mother tongue was English to ensure equivalence between the translations. Following this, a sample of 30 participants (15 participants who met the inclusion criteria of the study and 15 participants who were retrieved from the general population) was asked to fill in the pre-final version of the MEC10-IT in order to assess its items’ comprehensibility [42,43]. At this stage, no further adjustment was made (Table 1 and Appendix A).

### 2.2. Materials and Methods

#### 2.2.1. Sample Size Determination

Considering the aim of the present study, the sample size was set a priori according to the “*n:q* criterion”—where n is the number of participants and q is the number of (free) model parameters to be estimated [44,45,46]. A ratio of five participants for each parameter was, therefore, guaranteed: *N*_minimum_ = 250.

#### 2.2.2. Procedure

A cross-sectional research method was employed to test the factor structure of the MEC10-IT. 

A sample of inpatients with severe obesity who were recruited at the IRCCS Istituto Auxologico Italiano, Ospedale San Giuseppe, Verbania, Italy, within their first week of a one-month rehabilitation program for weight reduction was asked to fill in a survey comprising selected socio-demographic questions, as well as the Italian version of the MEC10-IT, of the modified Yale Food Addiction Scale 2.0 (mYFAS 2.0), and of the Binge Eating Scale (BES).

The inclusion criteria were as follows: (A) having Italian as their first language; (B) being 18 years old or older; (C) having a BMI ≥ 35 kg/m^2^; and (D) providing signed, written informed consent to participate in the study. The exclusion criteria were as follows: (A) the incapability to complete the survey due to vision and/or cognitive problems and (B) failure to provide answers to all items. All participants signed the written and informed consent. 

#### 2.2.3. Participants

A total of 474 participants were initially recruited—but 22 inpatients were left out due to missing answers. 

The final sample comprised 452 inpatients with severe obesity: 206 males (45.6%) and 246 females (54.4%), aged 18 to 82 years (*mean* = 54.18, *SD* = 12.657), with a BMI ranging from 35.08 to 86.18 kg/m^2^ (*mean* = 43.22, *SD* = 6.77).

### 2.3. Measures

#### 2.3.1. The Measure of Eating Compulsivity (MEC10)—Italian Version

The MEC10-IT is a self-report questionnaire composed of 10 items answered on a 5-point Likert-type response scale (from 0 = “*Very Untrue*” to 4 = “*Very True*”) assessing compulsive eating behaviors. High scores correspond to a high degree of eating compulsivity. In the first validation study [39], the MEC10 showed acceptable internal consistency, with Cronbach’s alpha being equal to 0.946.

#### 2.3.2. The Modified Yale Food Addiction Scale 2.0 (mYFAS2.0)

The mYFAS 2.0 [47,48] is a 13-item self-report questionnaire scored on an 8-point Likert-type scale (ranging from 0 = “*never*” to 7 = “*every day*”) assessing the presence of FA. Similarly to its original extended version (YFAS 2.0) [49,50,51], the mYFAS 2.0 evaluates the 11 DSM-5 diagnostic criteria for SRADs [28] and the impairment and/or distress related to food experience by the subjects over the previous 12 months—for example, (A) consumed more food than intended; (B) unable to cut down or stop; (C) use despite physical/emotional consequences; and (D) craving. The mYFAS2.0 offers the following two scoring options: the *symptom count* score (namely, the number of FA symptoms experienced in the previous year) and the *diagnostic score* (namely, the absence/presence of FA). In the present sample, the mYFAS2.0 showed satisfactory internal consistency, as the KR20 coefficient was 0.831.

#### 2.3.3. The Binge Eating Scale (BES)

The BES [52,53] is a self-report measure of binge eating severity in both general [54] and clinical populations [55]. It consists of 16 items related to 2 different dimensions (FC—feelings/cognitions; and B—behaviors), and a total score [55]. The BES has received general support as a reliable and valid measure of eating-related pathology—especially for BED. It commonly shows a satisfactory internal consistency, and several studies highlight its ability to discriminate between clinical and non-clinical individuals [52]. A total score equal to, or higher than, 16 (total BES ≥ 16) is considered a clinical cutoff for BED [52,53]. In the present sample, the BES showed satisfactory internal consistency, as Cronbach’s alphas were 0.890, 0.809, and 0.816 for the BES total score, the FC subscale, and the B dimension, respectively.

### 2.4. Statistical Analyses

The following R [56,57] packages were used: corrplot [58], lavaan [59,60], and pscl [61,62]; psych [63]; and psychTools [64], semTools [65], and tidyverse [66]. Graphics were carried out using semPlot package [67].

**Figure 1:**
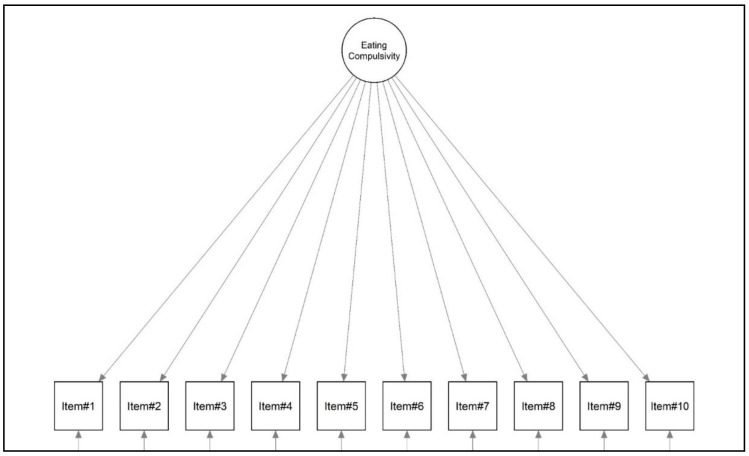
Study 1. Structural model of the Italian MEC.

A first-order unidimensional model with all 10 items of the MEC10-IT loading onto the single latent dimension ‘*eating compulsivity*’ was set (Figure 1). The diagonal weighted least square (DWLS) estimator was used to assess the factorial structure of the MEC10-IT [68,69,70,71,72]. The model fit was evaluated with the following fit indices: (A) the chi-square statistic (χ^2^), (B) the root mean square error of approximation (RMSEA), (C) the comparative fit index (CFI), and (D) the standardized root mean residual (SRMR) [68,69,70,72,73]. The cutoff criteria used to assess the goodness of fit were as follows: (A) statistical non-significance of the χ^2^, (B) an RMSEA lower than 0.08, (C) a CFI higher than 0.95, and (D) an SRMR lower than 0.08 [68,69,70,72,73].

The internal consistency of the tool was assessed with Cronbach’s alpha (α) and McDonald’s omega (ω) [74,75,76]. The convergent validity was measured with the Pearson correlation coefficient [77] and interpreted using Cohen’s benchmarks: *r* < 0.10, trivial; *r* from 0.10 to 0.30, small; *r* from 0.30 to 0.50, moderate; *r* > 0.50, large [78].

### 2.5. Results

#### 2.5.1. Structural Validity

The MEC10-IT showed an excellent fit to the data. The chi-square statistic resulted to be statistically significant: χ^2^ (35) = 93.125; and *p* < 0.001. The RMSEA was lower than the cutoff threshold: RMSEA = 0.061; 90%CI [0.046, 0.076]; and *p*(RMSEA < 0.05) = 0.113 *ns*. The CFI was lower than the cutoff threshold: CFI = 0.998. In addition, the SRMR was lower than the cutoff threshold: SRMR = 0.034.

As reported in Table 1, all of the items’ loadings were statistically significant and ranged from 0.766 (item#9) to 0.869 (item#4), with a mean that was equal to 0.824 and an *SD* equal to 0.033.

#### 2.5.2. Internal Consistency

The reliability analysis revealed satisfying results: Cronbach’s alpha = 0.939 and McDonald’s ω = 0.948.

#### 2.5.3. Convergent Validity

**Figure 2:**
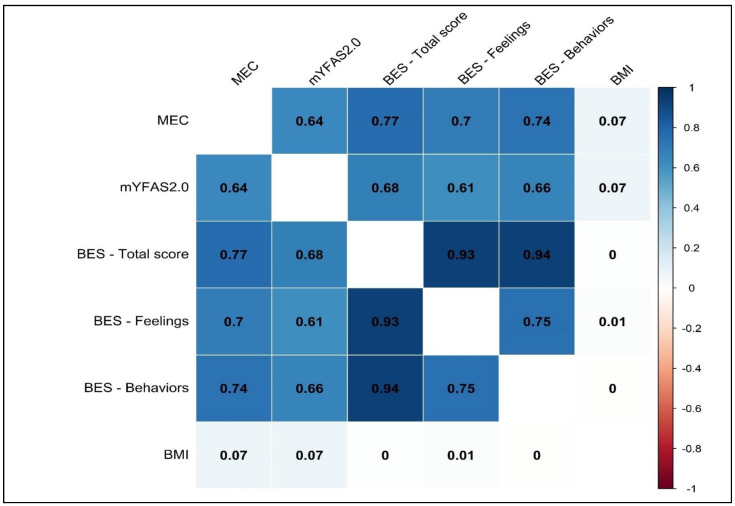
Study 1. Correlations between variables. *Note*: All *p*-values are less than 0.001; except for correlations related to BMI with all *r_s_’ p* > 0.050 *ns*.

The correlations between the MEC10-IT and the mYFAS2.0 symptom count (*r* = 0.644, *p* < 0.001), the BES total score scale (*r* = 0.767; *p* < 0.001), the BES FC subscale (*r* = 0.695; *p* < 0.001), and the BES B subscale (*r* = 0.738, *p* < 0.001) were moderate to large. A non-statistically significant (*r* = 0.041, *p* = 0.418 *ns*) correlation was, instead, found between the MEC10-IT and the participants’ BMI. The results are reported in Figure 2.

## 3. Study 2: In-Depth Analysis of the MEC10-IT

### 3.1. Materials and Methods

#### 3.1.1. Sample Size Determination

As in Study 1, the “*n:q* criterion” was used to calculate the minimum sample size [44,45,46], and a ratio of five participants for each parameter (5:1; *n*_minimum_ = 250) was ensured.

#### 3.1.2. Procedure

The inpatients with severe obesity (BMI ≥ 35 kg/m^2^) were selected for inclusion in this study based on the same procedures and criteria as those used in Study 1; however, this sample differs from that of Study 1. In addition, in line with previous investigations [50,79], a community sample was recruited in Padua, Italy, through personal invitations and advertisements. A snowball sampling technique was used.

The inclusion criteria were as follows: (A) being a native Italian speaker; (B) being 18 years old or older; and (C) providing signed, written informed consent to participate in the study. The participants were, instead, excluded if they presented with vision and cognitive impairments preventing them from filling in the questionnaires.

#### 3.1.3. Participants

The final sample of this study was composed of 764 participants, including 453 inpatients with severe obesity and a community sample of 311 participants. The clinical population comprised 196 males (43.3%) and 257 females (56.7%), in the age range from 18 to 87 years (*mean* = 53.46, *SD* = 12.973), and with a BMI ranging from 35.15 to 80.11 kg/m^2^ (*mean* = 43.237 kg/m^2^, *SD* = 6.649). The non-clinical sample included 88 males (28.3%) and 223 females (71.7%), in the age range from 18 to 82 years (*mean* = 31.53, *SD* = 15.747), and with a BMI ranging from 17.10 to 39.26 kg/m^2^ (*mean* = 21.77 kg/m^2^, *SD* = 3.283).

#### 3.1.4. Measures

The same survey that was used in Study 1 was administered to each participant, in addition to the Dutch Eating Behavioral Questionnaire (DEBQ).

The DEBQ [80,81] is a 33-item self-report measure of behaviors and attitudes related to eating disorders that is commonly used in both non-clinical [54,82] and clinical samples [83]. The responses are scored on a five-point Likert-type scale (ranging from 1 = “*never*” to 5 = “*very often*”) and loaded onto the following three dimensions: emotional eating (EE), restrained eating (RE), and external eating (ExE), in addition to providing a total score. In the present sample, the Cronbach’s alphas were 0.921, 0.883, 0.968, and 0.867 for the total score, the RE subscale, the EE subscale, and the ExE subscale, respectively. 

In the present sample, the MEC10-IT showed adquate internal consistency: Cronbach’s alphas was 0.944. Also, in this second study the mYFAS2.0 showed adquate internal consistency: Cronbach’s alphas was 0.917. Lastly, also the BES showed adquate internal consistency, as Cronbach’s alphas were 0.894, 0.807, and 0.823 for the BES total score, the FC subscale, and the B dimension, respectively.

#### 3.1.5. Statistical Analysis

The following R [56,57] packages were used: corrplot [58], ggplot2 [84], lavaan [59,60], plotROC [85], pROC [86], psych [63], psychTools [64], semTools [65], and tidyverse [66].

A first-order single-factor model (Figure 1) was specified for both the non-clinical and the clinical samples. The DWLS estimator was run [68,71]. The model fit was calculated with the above-mentioned model fit indices—the χ^2^, the RMSEA, the CFI, and the SRMR [68,73]. The following cutoff criteria for the goodness of fit were used: *p*(χ^2^) > 0.050 *ns*; RMSEA ≤ 0.08; CFI ≥ 0.95; and SRMR ≤ 0.08 [68,69,70,73]. The model structure was tested on each sample independently.

The measurement invariance analysis for the categorical data was run [87]. According to the guidelines [73,87,88], the following four (nested) models were set and their model parameters were consecutively forced to equality: configural invariance (Model 1: equal factorial structure); metric invariance (Model 2: equal factorial structure and item factor loadings); scalar invariance (Model 3: equal factorial structure, item factor loadings, and item thresholds); and means invariance (Model 4: equal factorial structure, item factor loadings, item thresholds, and latent means) [42,73,87,88,89,90,91]. These four models were sequentially compared. Model evaluations were performed by using the test differences in three fit indices, with the following criteria as cutoffs for model equality: DIFFTEST (equal to Δχ^2^; *p*-value > 0.050), ΔRMSEA (<0.015), and ΔCFI (<0.010). The overpass of these cutoffs by two out of the three indices—combined with worse fit indices—was considered evidence of model inadequacy [68,73,89,90,92,93].

The internal consistency of the tool was evaluated with Cronbach’s alpha (α). In addition, McDonald’s omega (ω) [74,75,76] was also used.

Moreover, the item discriminant power (IDP) was computed in order to evaluate the ability of the items to discriminate between participants with low or high EC [94,95]. In more detail, the maximum total score and the quartile rank for each participant were calculated. Then, independent sample *t*-tests—and their effect size (Cohen’s *d*) [78]—were computed to assess the IDP using the total score of the scale as the dependent variable and its lowest and highest quartile as the grouping variable [94,95]. In addition, the item–total correlation (adjusted) was computed [77,96,97].

The Pearson correlation coefficient was used to assess the convergent validity and was interpreted using Cohen’s benchmarks [77,78].

The incremental validity was also examined. A first regression analysis (generalized linear model with zero-inflated negative binomial distribution) was conducted to assess the increase in the explained variance (Δpseudo-*R*^2^) in the FA symptoms that were related to the MEC10-IT. A second multiple regression analysis (general linear model with continuous dependent variable) was conducted to assess the increase in the explained variance (Δ*R*^2^) in the binge eating tendencies that were related to the MEC10-IT. For each regression analysis, eating attitude measures were first entered into the regression equation (first block). The MEC10-IT total score was then added (second block) and the ΔR^2^ was checked in order to evaluate its contribution.

Moreover, receiver operating characteristics (ROC) curves were used to assess the accurateness of the MEC10-IT to distinguish (A) the participants presenting FA from those without FA; and (B) the participants with BED from those not presenting binge eating symptoms [98,99]. In accordance with previous studies [81], an overall sample of inpatients with severe obesity was used by merging the inpatients who were enrolled in Study 1 and the inpatients who were enrolled in Study 2. Thus, the final sample that was used to run the ROC curve analysis was equal to 909 participants. The overall accuracy–validity of the MEC10-IT was assessed with the area under the ROC curve (AUC; 5000 stratified bootstrap resamples). Swets’ benchmarks were used to interpret the AUC [100,101]. Furthermore, the accuracy (ACC), sensitivity (SE), and specificity (SP) were calculated for each MEC cutoff point [98,99].

### 3.2. Results

#### 3.2.1. Structural Validity

When combining the results of the two samples, the MEC10-IT presented a good fit to the data. The chi-square statistic was statistically significant: χ^2^ (35) = 176.414; and *p* < 0.001. Additionally, all of the other fit indices revealed a good fit to the data: the RMSEA = 0.073; 90%CI 0.062–0.084; *p*(RMSEA < 0.05) < 0.001; the CFI = 0.998; and the SRMR = 0.038.

In the sample of inpatients with severe obesity, the chi-square statistic was statistically significant: χ^2^ (35) = 101.238; and *p* < 0.001. Still, the RMSEA (RMSEA = 0.065; 90%CI: 0.050–0.080; *p*(RMSEA < 0.05) = 0.048), the CFI (0.998), and the SRMR (0.034) did indicate a good model fit. As reported in Table 2, all of the items’ loadings were statistically significant and ranged from 0.788 (item#1) to 0.874 (item#6) (*mean* = 0.836; *SD* = 0.031).

In the community sample, the chi-square statistic was statistically significant: χ^2^ (35) = 99.344; and *p* < 0.001. Still, the RMSEA (RMSEA = 0.077; 90%CI: 0.059–0.095; *p*(RMSEA < 0.05) = 0.007), the CFI (0.997), and the SRMR (0.051) did indicate a good model fit. As reported in Table 2, all of the items’ loadings were statistically significant and ranged from 0.737 (item#1) to 0.929 (item#8) (*mean* = 0.832; *SD* = 0.057).

#### 3.2.2. Psychometrics Properties

The IDP analysis showed that the 10 items of the MEC10-IT were able to discriminate among the participants with low and high levels of eating compulsivity in both of the samples (Table 2). As for the sample of inpatients with severe obesity, the discrimination parameter *t*_i_ ranged from |21.68| (item#1) to |33.66| (item#6), with an associated effect size (Cohen’s *d*) ranging from |2.92| to |4.49|, respectively. In the community sample, the discrimination parameter *t*_i_ ranged from |14.13| (item#10) to |25.01| (item#6), with an associated effect size (Cohen’s *d*) ranging from |2.24| to |3.98|, respectively. In addition, the item–total correlation (adjusted) revealed a moderate-to-strong association between each item of both of the samples and the MEC10-IT total score (Table 2).

The reliability analysis revealed satisfying results. In the sample of inpatients with severe obesity, Cronbach’s alpha was 0.943, and McDonald’s ω was 0.954, while among the general population, Cronbach’s alpha was 0.935, and McDonald’s ω was 0.953.

**Figure 3:**
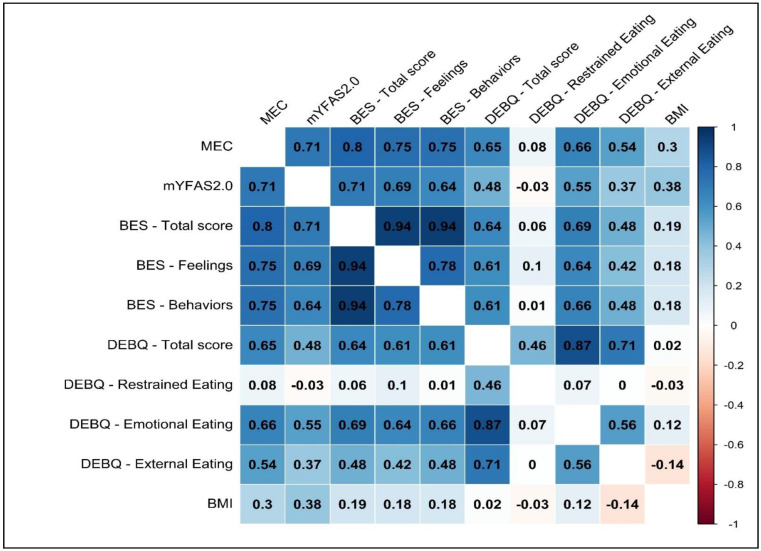
Study 2. Correlations among variables.

Considering the total sample—in line with Study 1—large correlations were observed between the MEC10-IT and the mYFAS2.0 symptom count (*r* = 0.714, *p* < 0.001), the BES total score (*r* = 0.796, *p* < 0.001), the BES FC (*r* = 0.751, *p* < 0.001), and the BES B (*r* = 0.749, *p* < 0.001). In addition, large correlations were detected between the MEC10-IT and the BEDQ total score (*r* = 0.649, *p* < 0.001), the DEBQ EE (*r* = 0.665, *p* < 0.001), and the DEBQ ExE (*r* = 0.540, *p* < 0.001). A non-statistically significant association was found between the MEC10-IT and the DEBQ RE (*r* = 0.077, *p* = 0.135, *ns*). Furthermore, the correlation between the MEC10-IT and the BMI was statistically significant (*r* = 0.298, *p* < 0.001). The results are shown in Figure 3.

#### 3.2.3. Measurement Invariance across Samples

*Configural Invariance*. The configural invariance model presented good model fit indices: χ^2^ (70) = 200.583, *p* < 0.001; the RMSEA = 0.070; the CFI = 0.998; and the SRMR = 0.041; signifying the MEC10-IT factor structure to be comparable between the clinical and non-clinical samples.

*Metric Invariance.* The metric invariance model well fitted the data: χ^2^ (79) = 283.614, *p* < 0.001; the RMSEA = 0.082; the CFI = 0.996; and the SRMR = 0.047. A statistically significant reduction in chi-square was detected: DIFTEST (9) = 83.031; and *p* < 0.001. Still, a non-statistically significant reduction in both RMSEA (|ΔRMSEA| = 0.012) and CFI (|ΔCFI| = 0.001) was observed—meaning that the items, irrespectively of the sample, were equally related to the latent factor.

*Scalar Invariance.* The scalar invariance model showed good model fit indices: χ^2^ (108) = 407.995, *p* < 0.001; the RMSEA = 0.085; the CFI = 0.995; and the SRMR = 0.041. A statistically significant chi-square reduction was observed: DIFTEST (29) = 124.38; and *p* < 0.001. Still, a non-statistically significant reduction in RMSEA (|ΔRMSEA| = 0.003) and CFI (|ΔCFI| = 0.002) was noticed. This indicates that the same expected item response at the same absolute level of the trait was obtained in both the clinical and the community samples.

*Latent Means Invariance.* The latent mean invariance model well fitted the data: χ^2^ (109) = 1145.008, *p* < 0.001; the RMSEA = 0.158; the CFI = 0.981; and the SRMR = 0.041. A statistically significant decline in chi-square was observed: DIFTEST (1) = 737.01; and *p* < 0.001. Still, a statistically significant reduction in both RMSEA (|ΔRMSEA| = 0.073) and CFI (|ΔCFI| = 0.013) was noticed—suggesting that the two samples had a different expected latent mean of the traits.

#### 3.2.4. Incremental Validity

The first multiple regression (zero-inflated negative binomial generalized linear model; ZINB GML) was performed in order to examine the contribution of the MEC10-IT to the explained variance of the mYFAS 2.0 symptom count (Table 3). Based on the results of the correlation analysis (Figure 3), in the first block, BES FC, BES B, DEBQ ExE, and DEBQ EE entered the regression model and accounted for 47.6% (Cragg and Uhler’s pseudo*R*^2^ = 0.476) of the explained variance of the mYFAS2.0 symptom count. In the second block, the MEC10-IT entered the regression equation and was found to significantly increase the proportion of the FA symptom count explained variance that was accounted by the model: 54.9% (Cragg and Uhler’s pseudo*R*^2^ = 0.549); Δpseudo*R*^2^ = 0.074.

The second multiple regression (linear model, LM) was performed in order to examine the contribution of the MEC10-IT to the explained variance in the BES total score (Table 3). Based on the results of the correlation analysis (Figure 3), in the first block, the mYFAS2.0 symptom count, DEBQ ExE, and DEBQ EE entered the regression and accounted for 65.3% (adjusted *R*^2^ = 0.653) of the BES total score. In the second block, the MEC10-IT entered the regression equation and was found to significantly increase the proportion of the explained variance of the binge eating tendencies that were accounted by the model: 72.2% (adjusted *R*^2^ = 0.722); Δ*R*_adj_^2^ = 0.069.

#### 3.2.5. Accuracy of the MEC10-IT as a Screening/Diagnostic Tool

**Figure 4:**
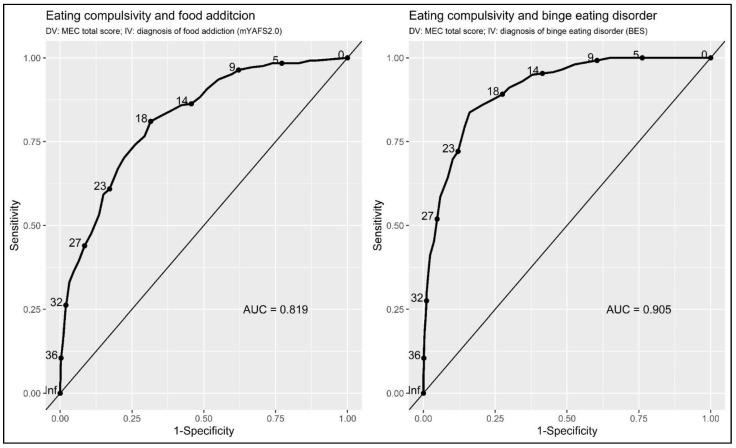
ROC curves.

The MEC10-IT highly accurately discriminated between the inpatients with severe obesity presenting with FA and those that did not present FA: AUC = 0.819; SE = 0.015; 95%CI = 0.789–0.849; and *p* < 0.001 (Figure 4—left panel). Considering a cutoff point of 17 (e.g., MEC ≥ 18: risk of FA), the ROC curves revealed an SE of 0.810 (95%CI: 0.762–0.859), an SP of 0.685 (95%CI: 0.649–0.720), and an ACC of 0.719 (95%CI: 0.719–0.720). Based on the mYFAS2.0 cutoffs, 657 (72.6%) of the participants were categorized as non-food-addicted, while 248 (27.4%) of the participants were considered to be food-addicted (total sample = 905). Therefore, when using a cutoff of 17 for the MEC10-IT, the ROC curves revealed that 450 (49.7%) of the participants were properly categorized as ‘true negative’ and 201 (22.2%) as ‘true positive’ (71.9%). In contrast, 47 (5.2%) of the respondents turned out to be ‘false negative’ and 207 (22.9%) of the participants turned out to be ‘false positive’ (28.1% misclassified).

The MEC10-IT also resulted in highly accurate discrimination between the inpatients with severe obesity presenting with BED and those without BED: AUC = 0.905; SE = 0.010; 95%CI = 0.884–0.925; and *p* < 0.001 (Figure 4—right panel). Considering a cutoff point of 20 (e.g., MEC ≥ 21: risk of BED), the ROC curves revealed an SE of 0.837 (95%CI: 0.792–0.882), an SP of 0.839 (95%CI: 0.811–0.868), and an ACC of 0.839 (95%CI: 0.838–0.839). Based on the BES, 647 (71.5%) of the participants were classified as non-binge-eaters, while 258 (28.5%) of the participants were classified as binge eaters (total sample = 905). Therefore, when using a cutoff of 20 for the MEC10-IT, the ROC curves displayed that 543 (60%) of the participants were properly categorized as ‘true negative’ and 216 (23.9%) as ‘true positive’ (83.87%). In contrast, 42 (4.6%) of the respondents turned out to be ‘false negative’ and 104 (11.5%) of the participants turned out to be ‘false positive’ (16.13% misclassified).

## 4. Discussion

Some individuals may show patterns of overeating, which exist on a continuum of severity ranging from casual indulgence to compulsive drive to consume certain foods [102] showing “addictive” tendencies toward highly processed foods similar to SRAD. In order to further understand the emerging concept of FA—and particularly the role played by its documented key component of compulsivity in the development and maintenance of obesity problems—the Brief Measure of Eating Compulsivity (MEC10) has been recently developed and tested on a sample of individuals with severe obesity in New Zealand, showing excellent psychometric proprieties [39].

Since this tool is not available for use in Italy, this study aimed to test the factorial structure of the Italian version of the MEC10 in a sample of Italian adult inpatients with severe obesity. In addition, this contribution had the purpose of offering an exhaustive examination of the psychometric properties of the questionnaire, which includes its measurement equivalence between clinical and non-clinical samples. Indeed, the comparability of the patient-reported outcome measures over different populations is essential to support clinical diagnostics, research on the quality of healthcare, and population health monitoring.

The results from the first study showed that, in a sample of adult inpatients with severe obesity, the single-factor structure of the MEC10-IT was satisfactory. In fact, the CFA showed excellent structural validity of the tool, and the reliability analysis was satisfying. Significant correlations were also found between the MEC10-IT total scores and both the mYFAS2.0 symptom count and the BES subscales, but not between the MEC10-IT and the individuals’ BMI. These findings support the association between the construct of FA and compulsive eating patterns as measured by the BES and are in line with those of previous studies that postulated the absence of a linear relationship between FA and BMI [50,103].

In Study 2, the structural validity and the reliability of the questionnaire were further confirmed in both clinical and non-clinical samples, and the MEC10-IT was shown to be a reliable tool in discriminating among the participants with low and high eating compulsivity across the populations. These results suggest the ability of the items to report inter-individual differences in the individuals’ compulsive behavior, as well as the ability of each item to represent its latent construct.

In the overall sample, large, significant positive correlations were also found between the MEC10-IT and both the mYFAS2.0 symptom count and the BED dimensions. In addition, the MEC10-IT was associated with the DEBQ total score, the DEBQ EE, the DEBQ ExE dimensions, and the individuals’ BMI, but not with the DEBQ RE. These findings suggest FA to be an interesting theoretical framework to examine disordered eating behaviors and highlight the important role of compulsive eating in the etiopathogenesis and maintenance of overweight and obesity and eating disturbances. Still, the constructs of FA and compulsivity that are related to BMI need to be clarified in future studies.

Because testing the invariance of factorial structures between different populations is central to scale validation, MI analysis was performed to explore this difference between the inpatients with severe obesity and the general population. The results show that the 10 items comprising the MEC10-IT were equally associated with the latent factor in each sample and that both of the populations had the equivalent expected item response at the identical absolute level of the trait. These findings suggest that the respondents in both of the samples had the same understanding of the MEC10-IT items (the factor structure was equivalent), with an equivalent strength (items were equally related to the latent construct), and with an equal baseline (item thresholds were equivalent). Still, the latent trait did not have the same distribution across the samples (latent means were different). This means that the MEC10-IT can be used for clinical and research purposes in both a population of individuals with obesity and a community sample (equal items threshold); however, the results should be interpreted with caution (different latent means) [104,105,106,107,108].

In addition, the results from the ROC analyses showed that the MEC10-IT represented a good screening/diagnostic measure for the detection of FA in individuals with severe obesity. In fact, it exhibited high accuracy (AUC = 0.819), sensitivity (0.810), and specificity (0.685) in distinguishing the inpatients with severe obesity presenting with FA from those that did not show FA symptoms. Equally, the MEC10-IT was shown to be reliable in identifying the presence of binge eating in people with severe obesity, as it was highly accurate (AUC = 0.905), sensible (0.837), and specific (0.839) in distinguishing between the inpatients with severe obesity with BED and those without BED. Furthermore, it has been shown that the MEC10-IT score accounted for unique variance in the mYFAS 2.0 symptom count dimension, as well as in BED tendencies.

Still, this study presents some limitations. First, the use of a cross-sectional research design did not permit the assessment of the probable changes in the MEC10-IT scores over time or its temporal stability. Future research should fill this gap by assessing the additional psychometric properties of the tool, including longitudinal MI and test–retest reliability. Moreover, the age of the participants largely varied in both of the samples. This may affect the individuals’ metabolism and compulsive eating behaviors. Future research should test possible explanatory models of compulsive eating behaviors by considering possible intervening variables (e.g., age, gender, BMI, etc.).

Regardless of the above-mentioned limitations, this contribution is the first aimed at investigating the psychometric proprieties of the MEC10-IT in both the general population and in individuals with severe obesity in Italy, confirming the reliability and the validity of the tools. The results of both of these studies are based, indeed, on solid and worldwide recommended statistics.

The MEC10-IT can, therefore, be used for clinical and research purposes to identify the presence of compulsive eating with accuracy and parsimony, thus representing a viable alternative to longer questionnaires, including the BES. Indeed, in the field of feeding disorders and EDs, eating compulsivity represents a crucial construct to be considered for more precise psychological interventions, as (1) it provides important information for both the conceptualization and the treatment of disordered eating patterns and (2) it may represent a transdiagnostic maintenance factor of EDs.

Moreover, compulsive eating behavior is a core element of either BED or FA diagnosis, but it is not required for obesity detection [109]. Still, 40–70% of individuals with BED and/or FA are likely to be overweight/obese [11,110,111,112]. Similarly, in adults with obesity and BED, an additional diagnosis of FA is associated with greater pathology and comorbid psychiatric disorders [113]. The high degree of comorbidity among these feeding and eating disorders supports their shared etiologies and/or underlying mechanisms, manifesting through compulsive eating behaviors. In addition, research suggests a partial overlap between FA, binge eating, and eating compulsivity—signifying that these three psychological constructs may be rather interconnecting [112]. Indeed, FA may lead to compulsive (over-) eating behaviors, and these—in turn—may become chronic to the point of determining markedly dysfunctional eating habits, such as binge eating behaviors—characterized by a loss of control and a reduced quality of life [112].

This study showed that the MEC10-IT might be a valid and reliable tool for the detection and measure of compulsive (over-) eating behaviors in individuals with severe obesity, as well as in the general population. Still, given that compulsive eating is present across weight classes, future studies should investigate if these findings are generalizable to a wider range of BMIs.

## 5. Conclusions

The MEC10-IT represents a reliable tool to assess the presence (and the level) of compulsive eating patterns in both clinical and non-clinical samples. In fact, the MEC10-IT showed good construct validity and reliability in both patients with severe obesity and in the general population. Still, compulsive eating behavior is characteristic of several eating-related conditions, including BED, obesity, and FA. This self-report questionnaire can therefore be used by clinicians and researchers to promptly assess the problem of the loss of control over the food that leads to obesity and eating-related pathologies above and beyond the already existing measures of FA and binge eating [114], thus informing the clinical prediction of compulsivity-related symptoms and supporting the development of interventions that are specifically aimed at addressing compulsive eating in obesity, BED and FA. Further research should seek to replicate these findings by employing cross-cultural and longitudinal designs and examine the MEC10-IT relationship of eating compulsivity with anthropometric and metabolic characteristics, as well as across psychopathological profiles.

## Figures and Tables

**Table 1:** Study 1. Item translation (English/*Italian*), descriptive statistics, and confirmatory factor analysis (CFA) results.

		Descriptive Statistics				CFA	
		Mean	SD	Sk	K	λ	*R* ^2^
1	I have urges to eat a lot of the time	1.111	1.160	0.709	−0.560	0.809	0.655
	*Sento il desiderio di mangiare per la maggior parte del tempo*						
2	I feel disturbed about my urges to eat	1.235	1.227	0.586	−0.858	0.778	0.605
	*Mi sento a disagio a causa del mio desiderio di mangiare*						
3	I have very little control over my eating	1.741	1.302	0.114	−1.161	0.828	0.686
	*Ho pochissimo controllo sul mio modo di mangiare*						
4	I often fear losing control of my eating	1.735	1.373	0.089	−1.309	0.869	0.756
	*Spesso temo di perdere il controllo sul mio modo di mangiare*						
5	I am not able to control how much I eat in the presence of any food	1.588	1.229	0.205	−1.015	0.847	0.718
	*In presenza di cibo, non sono in grado di controllare quanto mangio*						
6	I often feel out of control around certain foods	1.788	1.318	0.046	−1.192	0.816	0.666
	*Spesso mi sento fuori controllo in presenza di certi cibi*						
7	Food is like a drug to me	1.369	1.321	0.560	−0.871	0.838	0.702
	*Il cibo è come una droga per me*						
8	It worries me how little control I have over my eating	1.759	1.327	0.113	−1.164	0.851	0.724
	*Mi preoccupa quanto poco controllo io abbia sul mio modo di mangiare*						
9	When I come across a very tasty food I can’t stop thinking about it	1.973	1.293	−0.130	−1.085	0.766	0.587
	*Quando mi trovo davanti a un cibo molto gustoso, non riesco a smettere di pensarci*						
10	I feel defeated by food	1.529	1.302	0.282	−1.095	0.843	0.711
	*Mi sento sconfitto dal cibo.*						

Note: SD = standard deviation; SK = skewness; K = kurtosis; λ = factor loading; *R*^2^ = explained variance.

**Table 2:** Study 2. Item descriptive statistics and item psychometric properties.

**Inpatients with Severe Obesity**									
	**Descriptive Statistics**				**Items Psychometric Properties**			**CFA**	
	**Mean**	**SD**	**SK**	**K**	***t***	***d***	***r*_(it-tot)_**	**λ**	***R* ^2^**
Item#1	1.17	1.197	0.687	−0.548	−21.68	2.92	0.704	0.788	0.620
Item#2	1.30	1.299	0.546	−1.034	−26.75	3.59	0.720	0.792	0.627
Item#3	1.77	1.266	−0.004	−1.151	−29.45	3.92	0.780	0.848	0.719
Item#4	1.76	1.334	0.083	−1.239	−29.38	3.92	0.763	0.837	0.700
Item#5	1.64	1.268	0.182	−1.070	−29.47	3.95	0.800	0.866	0.750
Item#6	1.83	1.336	0.037	−1.237	−33.66	4.49	0.798	0.874	0.764
Item#7	1.42	1.342	0.481	−1.024	−29.55	3.97	0.798	0.870	0.758
Item#8	1.83	1.335	0.047	−1.190	−27.35	3.63	0.787	0.843	0.711
Item#9	2.04	1.315	−0.171	−1.088	−23.89	3.18	0.738	0.809	0.655
Item#10	1.58	1.375	0.369	−1.097	−28.39	3.79	0.766	0.833	0.694
**General Population**									
	**Descriptive Statistics**				**Items Psychometric Properties**			**CFA**	
	**Mean**	**SD**	**SK**	**K**	***t***	***d***	***r*_(it-tot)_**	**λ**	***R* ^2^**
Item#1	1.18	1.026	0.527	−0.616	−15.76	2.52	0.650	0.737	0.543
Item#2	0.79	1.081	1.264	0.615	−15.94	2.52	0.758	0.861	0.742
Item#3	0.99	1.063	1.020	0.438	−15.68	2.49	0.689	0.770	0.592
Item#4	1.13	1.261	0.776	−0.658	−23.62	3.75	0.743	0.836	0.698
Item#5	1.03	1.003	0.786	0.064	−21.81	3.48	0.790	0.866	0.750
Item#6	1.25	1.226	0.575	−0.843	−25.01	3.98	0.742	0.825	0.680
Item#7	0.98	1.151	1.040	0.156	−17.70	2.80	0.769	0.857	0.735
Item#8	0.77	1.097	1.383	0.995	−18.35	2.90	0.831	0.929	0.863
Item#9	1.38	1.214	0.458	−0.772	−20.48	3.27	0.696	0.778	0.606
Item#10	0.68	1.080	1.517	1.316	−14.13	2.24	0.746	0.864	0.746

*Notes*: SD = standard deviation; SK = skewness; K = kurtosis; IDP = item discriminant power; *t* = *t*-test; *d* = Cohen’s d; *r*_(it-tot)_ = item–total correlation (adjusted); λ = factor loading; *R*^2^ = explained variance.

**Table 3:** Study 2. Incremental validity.

**First Regression Analysis (ZINB GML)—Dependent Variable: mYFAS2.0 Symptom Count**							
	**Predictors**	**β**	**se**	***z***	***p*-Value**	**pseudo*R*^2^**	**Δpseudo*R*^2^**
Block 1	BES FC_(CM)_	0.080	0.017	4.770	<0.001		
	BES B_(CM)_	0.017	0.015	1.152	0.249		
	DEBQ EE_(CM)_	0.116	0.079	1.467	0.142		
	DEBQ ExE_(CM)_	0.101	0.065	1.560	0.119		
	BES FC_(ZI)_	−0.188	0.065	−2.898	0.003		
	BES B _(ZI)_	−0.145	0.072	−1.995	0.046		
	DEBQ EE _(ZI)_	0.602	0.311	1.932	0.053		
	DEBQ ExE _(ZI)_	−0.401	0.243	−1.652	0.098	0.476	
Block 2	BES FC_(CM)_	0.053	0.016	3.283	0.001		
	BES B_(CM)_	0.008	0.014	0.553	0.580		
	DEBQ EE_(CM)_	0.094	0.060	1.557	0.119		
	DEBQ ExE_(CM)_	−0.007	0.078	−0.095	0.924		
	MEC_(CM)_	0.030	0.007	4.135	0.001		
	BES FC_(ZI)_	−0.098	0.071	−1.374	0.169		
	BES B_(ZI)_	−0.018	0.071	−0.260	0.795		
	DEBQ EE_(ZI)_	−0.185	0.275	−0.673	0.501		
	DEBQ ExE_(ZI)_	0.870	0.344	2.527	0.011		
	MEC_(ZI)_	−0.144	0.031	−4.672	<0.001	0.549	0.074
**Second Regression Analysis (LM)—Dependent Variable: Binge Eating Tendencies (BES)**							
	**Predictors**	**β**	**se**	***t***	***p*-Value**	***R*_adj_^2^**	**Δ*R*_adj_^2^**
Block 1	DEBQ EE	3.033	0.332	9.143	<0.001		
	DEBQ ExE	1.085	0.430	2.523	0.012		
	mYFAS2.0	1.608	0.120	13.385	<0.001	0.653	
Block 2	DEBQ EE	1.970	0.316	6.230	<0.001		
	DEBQ ExE	0.071	0.399	0.179	0.858		
	mYFAS2.0	0.923	0.128	7.184	<0.001		
	MEC	0.367	0.038	9.708	<0.001	0.722	0.069

Note: ZINB GLM = zero-inflated negative binomial generalized linear model; LM = linear model; (…)_(CM)_ = coefficients for the count model; (…)_(ZI)_ = coefficients for the zero-inflated model; β = unstandardized estimate; se = standard error; *z* = *z*-value; *t* = *t*-value; pseudo*R*^2^ = Cragg and Uhler’s pseudo R-squared (equal to Nagelkerke’s pseudo*R*^2^); *R*_adj_^2^ = adjusted R-squared.

## Data Availability

The data presented in this study are available on request from the corresponding author. The data are not publicly available due to privacy restrictions.

## Appendix A

MEC10-IT

**ISTRUZIONI:** La preghiamo di rispondere a tutte le domande ponendo un segno in corrispondenza del valore numerico—compreso tra 0 (COMPLETAMENTE FALSO) e 4 (COMPLETAMENTE VERO)—che meglio La rappresenta.

**NON ESISTONO RISPOSTE GIUSTE O SBAGLIATE.**

	**0** COMPLETAMENTE FALSO	**1** FALSO	**2** NÉ FALSO, NÉ VERO	**3** VERO	**4** COMPLETAMENTE VERO				
**1**	Sento il desiderio di mangiare per la maggior parte del tempo.				**0**	**1**	**2**	**3**	**4**
**2**	Mi sento a disagio a causa del mio desiderio di mangiare.				**0**	**1**	**2**	**3**	**4**
**3**	Ho pochissimo controllo sul mio modo di mangiare.				**0**	**1**	**2**	**3**	**4**
**4**	Spesso temo di perdere il controllo sul mio modo di mangiare.				**0**	**1**	**2**	**3**	**4**
**5**	In presenza di cibo, non sono in grado di controllare quanto mangio.				**0**	**1**	**2**	**3**	**4**
**6**	Spesso mi sento fuori controllo in presenza di certi cibi.				**0**	**1**	**2**	**3**	**4**
**7**	Il cibo è come una droga per me.				**0**	**1**	**2**	**3**	**4**
**8**	Mi preoccupa quanto poco controllo io abbia sul mio modo di mangiare.				**0**	**1**	**2**	**3**	**4**
**9**	Quando mi trovo davanti a un cibo molto gustoso, non riesco a smettere di pensarci.				**0**	**1**	**2**	**3**	**4**
**10**	Mi sento sconfitto dal cibo.				**0**	**1**	**2**	**3**	**4**
